# Endoscopic biopsy techniques in Barrett esophagus patients: a multidesign study

**DOI:** 10.1055/a-2606-7682

**Published:** 2025-06-24

**Authors:** Ilse N. Beaufort, Sjoerd G. Elias, Elisabeth M.P. Akkerman, Anya N. Milne, Lodewijk A. A. Brosens, Marc A. M. T. Verhagen, Lorenza Alvarez Herrero, Bas L. A. M. Weusten

**Affiliations:** 1 Department of Gastroenterology and Hepatology, University Medical Center Utrecht, Utrecht, Netherlands; 2 Department of Gastroenterology and Hepatology, St. Antonius Hospital, Nieuwegein, Netherlands; 3 Julius Center for Health Sciences and Primary Care, University Medical Center Utrecht, Utrecht, Netherlands; 4 Department of Pathology, St. Antonius Hospital, Nieuwegein, Netherlands; 5 Department of Pathology, University Medical Center Utrecht, Utrecht, Netherlands; 6 Department of Gastroenterology and Hepatology, Diakonessenhuis, Utrecht, Netherlands

## Abstract

**Background:**

The impact of different random biopsy techniques for Barrett esophagus (BE) surveillance on histopathological quality is unclear. We compared the double- vs. single-biopsy method and advance-and-close vs. turn-and-suction technique.

**Methods:**

In a multicenter, factorial design trial (Part I), BE patients were randomly assigned to the double- or single-biopsy method and advance-and-close or turn-and-suction technique (1:1:1:1). In a before–after study (Part II), the optimal biopsy method and technique were implemented in clinical practice. The primary end point in both parts was biopsy size.

**Results:**

In Part I (107 patients, 1024 biopsies), single-method biopsies were 25% larger than double-method biopsies (3.34 mm ^2^ [95%CI 3.10–3.57] vs. 2.68 mm ^2^ [95%CI 2.45–2.92]; P < 0.001). Mean (95%CI) biopsy size was 2.95 mm ^2^ (2.72–3.19) and 3.08 mm ^2^ (2.85–3.31) with advance-and-close and turn-and-suction techniques, respectively (P = 0.44). The interaction term between the co-primary comparisons was P = 0.08. Mean (95%CI) biopsy size for double-biopsy + advance-and-close, double-biopsy + turn-and-suction, single-biopsy + advance-and-close, and single-biopsy + turn-and-suction was 2.77 mm ^2^ (2.44–3.09), 2.61 mm ^2^ (2.29–2.93), 3.14 mm ^2^ (2.81–3.46), and 3.54 mm ^2^ (3.22–3.86), respectively. In Part II, 46 and 44 patients were included before and after implementation of the single-biopsy method and turn-and-suction technique, in whom this combination was used in 16/46 (35%) and 44/44 (100%) patients, respectively. Mean (95%CI) biopsy size increased by 18%, from 3.31 mm ^2^ (2.95–3.68) to 3.90 mm ^2^ (3.50–4.29; P = 0.03).

**Conclusion:**

BE surveillance biopsies should be taken with the single-biopsy method and turn-and-suction technique to increase biopsy size.

**Conclusion:**

BE surveillance biopsies should be taken with the single-biopsy method and turn-and-suction technique to increase biopsy size.

## Abstract (de)

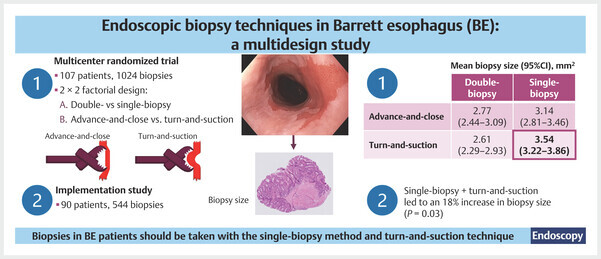

## Introduction

The goal of endoscopic surveillance in Barrett esophagus (BE) is early detection of dysplasia and esophageal adenocarcinoma. As dysplastic lesions in BE can be subtle and easily overlooked, obtaining four-quadrant random biopsies every 2 cm of BE length is still considered an essential part of BE surveillance endoscopies 1 2 3 4 .

Meticulous histopathological assessment of random biopsies is essential, considering endoscopic follow-up and treatment of patients with BE mainly depend on pathology results. High-quality biopsies, that is, biopsies that are large, well-oriented, without crush artifacts, and representing all mucosal layers, may facilitate histopathological assessment.

Random biopsies in BE surveillance can be obtained using different methods and techniques. Endoscopists may use the “single-biopsy” method or the “double-biopsy” method, in which one or two biopsy specimen per passage of the biopsy forceps are taken, respectively. In addition, the “turn-and-suction technique” or the “advance-and-close” technique can be used 5 . When using the turn-and-suction technique, the biopsy forceps is inserted through the instrument channel of the endoscope, after which the forceps cups are opened. The next steps involve withdrawing the opened cups against the tip of the endoscope, turning the endoscope towards the target area and suctioning air from the lumen, which causes the mucosa to collapse into the cups. In contrast, the advance-and-close technique requires passage of the biopsy forceps through the instrument channel of the endoscope and beyond the endoscope tip into the lumen, after which the opened forceps cups are pressed into the mucosa 5 .

The effect of these different biopsy sampling methods and techniques on histopathological quality of BE biopsies is currently unclear. We therefore aimed to compare the double- vs. single-biopsy method and the advance-and-close vs. turn-and-suction technique in a multidesign study.

## Methods

This study consisted of three different parts: an exploratory prestudy, a two-by-two factorial design randomized trial (Part I), and an uncontrolled before–after clinical implementation study (Part II).

The exploratory prestudy involved a retrospective cohort analysis to compare BE biopsies obtained by endoscopists with different levels of expertise in BE surveillance care (i.e. dedicated BE endoscopists and nondedicated BE endoscopists). This study aimed to evaluate whether differences in biopsy methods and techniques were associated with differences in biopsy size, regardless of the endoscopists’ level of expertise in BE surveillance care. This prestudy was performed in one teaching hospital (St. Antonius Hospital) between October 2019 and March 2021. Further methods and results are discussed in the online-only Supplementary material ( **Supplementary Methods and Results** and **Table 1s** ).

Part I was a two-by-two factorial design randomized trial to compare different biopsy methods and techniques in BE patients, obtained by dedicated BE endoscopists and performed in one teaching hospital and one university clinic (St. Antonius Hospital and University Medical Center Utrecht) between June 2021 and December 2022. The two-by-two factorial design was chosen for efficiency reasons.

Part II was an uncontrolled before–after study performed in a community hospital (Diakonessenhuis) to compare biopsies obtained by nondedicated BE endoscopists before (January 2021 – November 2022) and after (December 2022 – January 2024) the implementation of the optimal biopsy method and technique determined in Part I.

The study protocol was approved by the institutional review boards of the participating hospitals. In the exploratory prestudy, electronic patient files were checked for registration of objection to participation in research, while written consent was obtained from all participants for Part I and Part II.

### Part I – two-by-two factorial design trial

#### Participants

All adult patients with a diagnosis of BE scheduled for surveillance endoscopy with random biopsies were eligible to participate. Patients with contraindications to biopsy and patients with ultralong BE segments (i.e. maximum length ≥10 cm) were excluded.

#### Interventions

Different biopsy techniques in Barrett esophagus.Video 1

Eligible patients were randomized into one of four study arms depending on biopsy method and technique:

1. single-biopsy method + advance-and-close technique
2. single-biopsy method + turn-and-suction technique
3. double-biopsy method + advance-and-close technique
4. double-biopsy method + turn-and-suction technique.

**Fig. 1:**
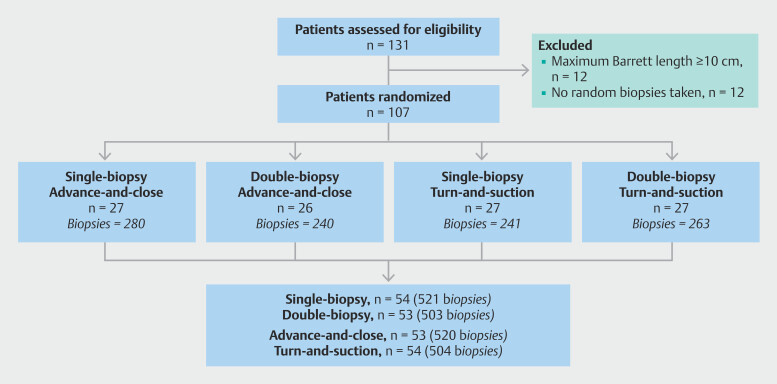
Patient flow diagram depicting the randomization process of study Part I (i.e. prospective factorial design trial).

The different biopsy techniques are demonstrated in Video 1 and schematically illustrated in **Fig. 1s** . Endoscopies were performed by two dedicated BE endoscopists (L.A.H. and B.L.A.M.W.). Random biopsy specimens were collected every 2 cm of BE length, similarly to standard practice. When a biopsy specimen was missing in the biopsy container because of dislodgement, an additional biopsy was taken in a separate container. The total number of biopsies lost in this way was recorded. The time required to obtain random biopsies was also recorded and defined as the time between the first insertion of the biopsy forceps until the last biopsy specimen had reached the pathology jar. Targeted biopsies were collected in separate containers and excluded from analyses. A research fellow or nurse practitioner was present during each endoscopy to ensure adequate data collection.

Two different biopsy forceps were used depending on the hospital site: Endo Jaw FB220U (Olympus, Tokyo, Japan) at St. Antonius Hospital and Radial Jaw 4 (Boston Scientific, Marlborough, Massachusetts, USA) at University Medical Center Utrecht.

#### Histopathological assessment

All biopsies were assessed regarding the following predefined parameters: 1) biopsy size, defined as the surface area in mm ^2^ ; 2) biopsy depth, defined as presence or absence of muscularis mucosae; 3) presence of crush artifacts, defined as present or absent; 4) biopsy orientation, defined as sufficient or insufficient.

Biopsy size was assessed by a member of the research team (I.N.B.), while all other parameters were assessed by two dedicated gastrointestinal pathologists (A.N.M, L.A.A.B). All assessments were completed in a blinded fashion.

#### Sample size

The sample size calculation was based on a subset of biopsies from the exploratory prestudy in which 66 biopsies were analyzed with respect to biopsy size. All biopsies were assumed to be taken with the double-biopsy method and advance-and-close technique. The mean surface area of biopsies was 3.106 mm ^2^ (SD 1.540). An increase in biopsy size of at least 15% (i.e. 0.466 mm ^2^ ) caused by either the single-biopsy method or the turn-and-suction technique was considered a relevant result. The sample size calculation was performed based on a two-way analysis of variance (“pwr2” package, R version 3.51. for Windows; R Foundation for Statistical Computing, Vienna, Austria), after which we corrected for the clustering of multiple biopsies per patient using the Design Effect (assuming a mean of 8 biopsies per patient and an intraclass correlation coefficient of 0.25). To demonstrate a 15% increase in biopsy size using a two-by-two factorial randomized design, the calculated sample size in each arm was 240 biopsies, resulting in a total sample size of 960 biopsies (power 80%, two-sided alpha 0.05, assuming no interaction between co-primary comparisons). This was translated to approximately 120 patients assuming a mean of 8 biopsies per procedure, but it was decided to continue recruiting participants until 240 biopsies per randomization arm were included.

#### Randomization

Patients were randomized in a 1:1:1:1 ratio over the four different study arms. Random permuted block randomization was used with block sizes of 4 and 8, stratified on BE length (≤3 cm or >3 cm) and hospital site. Randomization was performed by research nurses using the REDCap randomization module 6 .

### Part II – uncontrolled before–after study

Similarly to Part I of our study, adult patients with a diagnosis of BE scheduled for surveillance endoscopy with random biopsies were eligible to participate. Exclusion criteria were patients with contraindications to biopsy and those with ultralong (≥10 cm) BE segments. No formal sample size calculation was performed; all eligible patients willing to participate were included during the aforementioned time frames.

Following the results of Part I, the combination of optimal biopsy method and technique for BE biopsies was implemented in routine clinical practice. Endoscopists received instructions from a dedicated BE endoscopist (B.L.A.M.W.) prior to implementation. Biopsy size, defined as surface area in mm ^2^ , was assessed by one member of the research team (I.N.B.) in an unblinded fashion. The same biopsy forceps (Radial Jaw 4; Boston Scientific) was used during the entire study period.

### Study outcomes

**Fig. 2:**
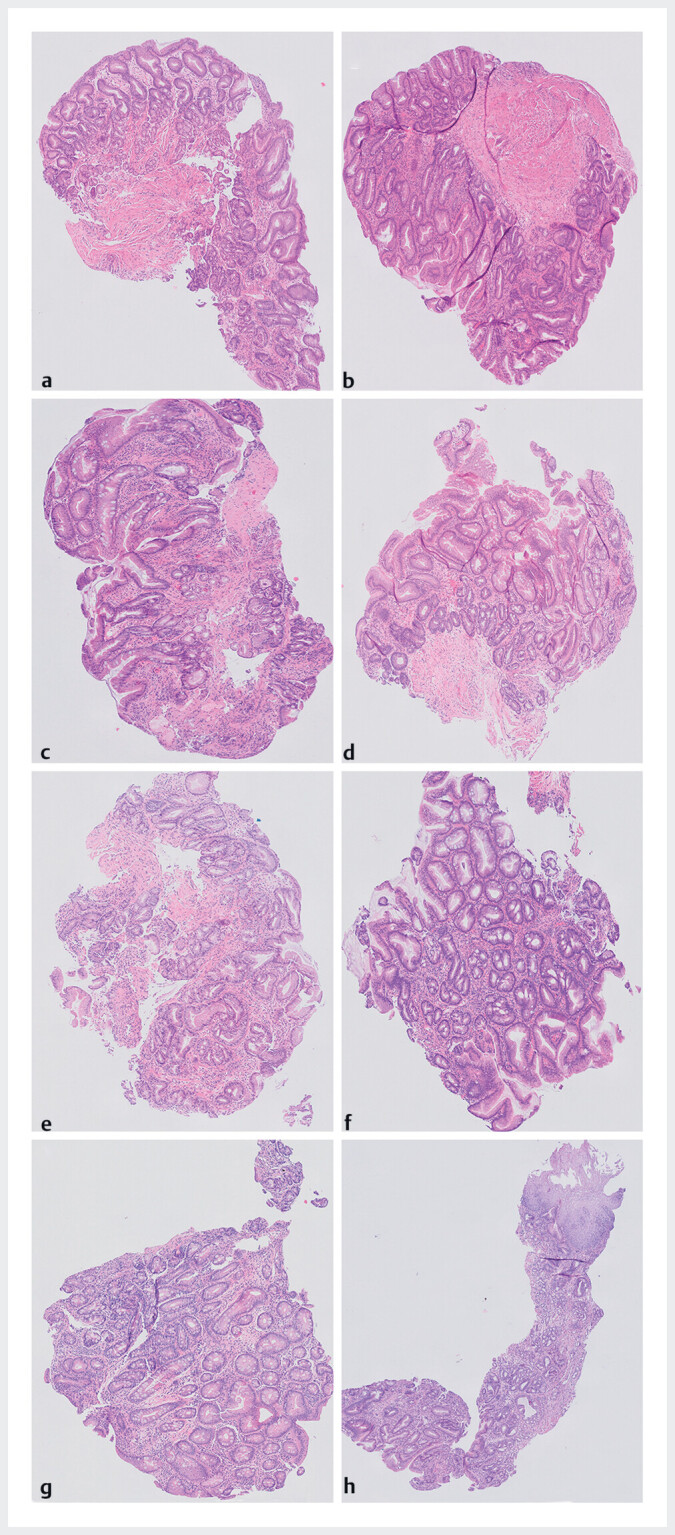
Examples of biopsies taken from Barrett esophagus mucosa using different methods and techniques. **a,b** Single-biopsy method and turn-and-suction technique. **c,d** Single-biopsy method and advance-and-close technique. **e,f** Double-biopsy method and turn-and-suction technique. **g,h** Double-biopsy method and advance-and-close technique.

The primary end point in both study parts was the size of biopsy specimens in mm ^2^ . Predefined secondary outcomes in Part I were: 1) the percentage of biopsies containing muscularis mucosae ( **Fig. 2s** ); 2) the percentage of biopsies without crush artifacts; 3) the percentage of well-oriented biopsies; 4) biopsy procedure time per biopsy; 5) the number of lost biopsy specimens.

### Biopsy specimen preparation and surface area assessment

All biopsies were fixed in formalin and subsequently processed and digitalized according to routine clinical care (Philips IntelliSite Image Management System at St. Antonius Hospital and Diakonessenhuis, or Sectra Digital Pathology PACS at University Medical Center Utrecht). The digital pathology programs contained a validated tool for surface area measurements. A single pathology slide per biopsy was generally digitalized at St. Antonius Hospital and Diakonessenhuis, meaning the digital pathology slide used for study purposes was the same slide used in clinical practice. Six slides per biopsy were routinely digitalized at University Medical Center Utrecht. Therefore, the first slide per biopsy was consistently used for pathological assessment during this study.

### Statistical methods

In both study parts, mixed-effects linear regression analyses (identity link function; restricted maximum likelihood estimation) with a random intercept per patient were used to assess differences in biopsy size. In Part I, fixed effects included biopsy method, biopsy technique, and stratification factors (i.e. BE length and hospital site), while biopsy method, biopsy technique, and BE length were included as fixed effects in Part II. Mean estimates with 95%CIs were derived from these models. Degrees of freedom, *P* values, and 95%CIs were derived using the Satterthwaite approximation, Wald test, and Wald CIs, respectively. Assumptions inherent to the statistical models were checked.

Additional mixed-effects logistic regression analyses (logit link function; restricted maximum likelihood estimation) including a random intercept per patient were used in Part I to evaluate differences in the presence of muscularis mucosae, presence of crush artifacts, biopsy orientation, and number of lost biopsies. Biopsy procedure time per biopsy was the only secondary end point that was compared at patient level, for which we used ordinary linear regression analyses.

In Part I, the co-primary comparisons were the two biopsy methods (i.e. the single-biopsy vs. double-biopsy method) and biopsy techniques (i.e. the advance-and-close vs. the turn-and-suction technique). We assumed no interaction between these co-primary comparisons; however, in additional analyses we tested for the significance of the interaction term between the co-primary comparisons of all outcomes (maximum likelihood estimation).

Data were analyzed in R version 3.5.1 for Windows. The significance level was set at *P* <0.05 without multiplicity adjustment.

## Results

### Part I – two-by-two factorial design randomized trial

#### Patients

A total of 107 patients were randomized, with baseline characteristics shown in Table 1 .

**Table:** **Table 1** Baseline characteristics of the included patients with Barrett esophagus undergoing surveillance endoscopies shown by randomization arm (study Part I; prospective factorial design trial). All patients were included at two Dutch teaching hospitals between June 2021 and December 2022.

	Total	Double-biopsy		Single-biopsy	
		Advance-and-close	Turn-and-suction	Advance-and-close	Turn-and-suction
Patients, n	107	26	27	27	27
Age, median (IQI), years	70 (64–74)	71 (65–73)	70 (62–75)	71 (67–74)	68 (62–73)
Male, n (%)	82 (77)	21 (81)	18 (67)	21 (78)	22 (81)
ASA classification, n (%)					
I	24 (22)	5 (19)	6 (22)	7 (26)	6 (22)
II	67 (63)	19 (73)	16 (59)	17 (63)	15 (56)
III	15 (14)	2 (8)	4 (15)	3 (11)	6 (22)
Unknown	1 (1)	0 (0)	1 (4)	0 (0)	0 (0)
Hospital site ^1^ , n (%)					
St. Antonius Hospital	75 (70)	18 (69)	19 (70)	19 (70)	19 (70)
UMC Utrecht	32 (30)	8 (31)	8 (30)	8 (30)	8 (30)
Endoscopist, n (%)					
Endoscopist 1	84 (79)	20 (77)	21 (78)	23 (85)	20 (74)
Endoscopist 2	23 (21)	6 (23)	6 (22)	4 (15)	7 (26)
Hiatal hernia, median (IQI), cm	4 (2–6)	4 (3–6)	4 (3–6)	3 (2–5)	3 (2–6)
BE length, median (IQI), cm					
Circumferential	1 (0–4)	3 (0–4)	1 (0–5)	1 (0–5)	1 (0–3)
Maximum	4 (2–7)	5 (2–6)	4 (2–7)	5 (2–7)	3 (2–5)
Maximum BE length ^1^ , n (%)					
≤3 cm	47 (44)	11 (42)	12 (44)	12 (44)	12 (44)
>3 cm	60 (56)	15 (58)	15 (56)	15 (56)	15 (56)
Esophagitis, n (%)	7 (7)	2 (8)	2 (7)	2 (7)	1 (4)
Sedation, n (%)					
No sedation	12 (11)	5 (19)	3 (11)	3 (11)	1 (4)
Midazolam	61 (57)	12 (46)	16 (59)	19 (70)	14 (52)
Propofol	34 (32)	9 (35)	8 (30)	5 (19)	12 (44)
ASA, American Society of Anesthesiologists; BE, Barrett esophagus; IQI, interquartile interval; UMC, University Medical Center. ^1^ Dichotomous hospital site (St. Antonius Hospital and UMC Utrecht) and BE length (≤3 cm and >3 cm) were used for stratified randomization.					

On average, 10 biopsies were taken per procedure (SD 5). Overall, 521 biopsies were obtained using the single-biopsy method and 503 using the double-biopsy method, while 520 biopsies were obtained with the advance-and-close technique and 504 with the turn-and-suction technique ( Fig. 1 , Fig. 2 ).

#### Biopsy size

The overall mean biopsy size was 3.02 mm ^2^ (95%CI 2.81 to 3.23). The distribution of biopsy sizes for biopsy methods and techniques are depicted in **Fig. 3s** and **Fig. 4s** , respectively. As shown in Table 2 , biopsies obtained using the single-biopsy method were significantly larger than those obtained with the double-biopsy method (3.34 mm ^2^ [95%CI 3.10 to 3.57] vs. 2.68 mm ^2^ [95%CI 2.45 to 2.92]) with a mean difference of 0.65 mm ^2^ (95%CI 0.33 to 0.98; *P* <0.001). In contrast, no significant difference in biopsy size was observed in biopsies taken with the turn-and-suction technique compared with the advance-and-close technique (3.08 mm ^2^ [95%CI 2.85 to 3.31] vs. 2.95 mm ^2^ [95%CI 2.72 to 3.19]), with a mean difference of 0.13 mm ^2^ (95%CI –0.20 to 0.45; *P* = 0.44). The interaction term between the co-primary comparisons was nonsignificant ( *P* = 0.08; with an additional 0.56 mm ^2^ [95%CI –0.07 to 1.19] if the single-biopsy method was combined with the turn-and-suction technique).

**Table:** **Table 2** Primary and secondary end points for the co-primary comparisons (i.e. double-biopsy method vs. single-biopsy method and advance-and-close technique vs. turn-and-suction technique) of study Part I (i.e. prospective factorial design trial). All values represent mean estimates and main effects derived from linear or logistic (mixed-effects) regression models corrected for stratification factors and if applicable, repeated measurements.

	Biopsy method				Biopsy technique			
	Double-biopsy, mean (95%CI) n = 503	Single-biopsy, mean (95%CI) n = 521	Single vs. double (ref) ^1^ , mean difference/OR (95%CI) ^2^	*P* ^3^	Advance-and-close, mean (95%CI) n = 520	Turn-and-suction, mean (95%CI) n = 504	Turn-and-suction vs advance-and-close (ref) ^1^ , mean difference/OR (95%CI) ^2^	*P* ^3^
Primary end point								
Biopsy size, mm ^2^	2.68 (2.45 to 2.92)	3.34 (3.10 to 3.57)	0.65 (0.33 to 0.98)	<0.001	2.95 (2.72 to 3.19)	3.08 (2.85 to 3.31)	0.13 (–0.20 to 0.45)	0.44
Secondary end points								
Histopathological parameters. %								
Presence of muscularis mucosae	56 (49 to 63)	62 (55 to 68)	1.26 (0.86 to 1.86) ^4^	0.24	57 (50 to 64)	61 (54 to 68)	1.14 (0.77 to 1.69) ^4^	0.50
Absence of crush artifacts	96 (93 to 98)	98 (95 to 99)	1.70 (0.78 to 3.72) ^5^	0.19	97 (95 to 99)	97 (94 to 98)	0.89 (0.41 to 1.93) ^5^	0.76
Well-orientated biopsies	71 (64 to 77)	81 (75 to 86)	1.74 (1.08 to 2.78)	0.02	78 (72 to 84)	77 (67 to 80)	0.77 (0.48 to 1.23)	0.28
Endoscopic parameters								
Biopsy time ^6^ , seconds	26 (24 to 28)	28 (26 to 30)	2 (–1 to 4)	0.26	24 (22 to 25)	31 (29 to 32)	7 (4 to 10)	<0.001
Retained and lost biopsies, %
Retained	94 (89 to 97)	99.7 (99 to 100)			99 (97 to 100)	98 (96 to 99)		
Lost	6 (3 to 11)	0.3 (0.07 to 1)	18.1 (4.18 to 78.4)	< 0.001	1 (0.5 to 4)	2 (0.6 to 4)	0.78 (0.35 to 1.72)	0.54
BE, Barrett esophagus. ^1^ In all models, biopsy method, biopsy technique, and stratification factors (i.e. BE length and study site) were included. No interaction was assumed between the co-primary comparisons. ^2^ Data are mean difference for biopsy size and biopsy time, and OR for histological parameters and retained/lost biopsies. ^3^ Derived from multiple linear or logistic (mixed-effects) regression models. ^4^ Muscularis mucosae present vs. absent. ^5^ Crush artifacts absent vs. present. ^6^ Defined as biopsy time (in seconds) per biopsy, assessed at patient level using ordinary linear regression.								

Biopsy sizes for each randomization group are presented in Table 3 . Among single-method biopsies, those obtained with the turn-and-suction technique were 13% larger than those obtained with the advance-and-close technique. However, this difference failed to reach statistical significance (mean biopsy size 3.54 mm ^2^ [95%CI 3.22 to 3.86] vs. 3.14 mm ^2^ [95%CI 2.81 to 3.46], respectively; *P* = 0.08).

#### Histopathological parameters

The muscularis mucosae was present in 56% (95%CI 49 to 63) of biopsies obtained using the double-biopsy method compared with 62% (95%CI 55 to 68) of biopsies taken with the single-biopsy method (odds ratio [OR] 1.26 [95%CI 0.86 to 1.86]; *P* = 0.24). Similarly, biopsies taken with the advance-and-close-technique and turn-and-suction technique contained muscularis mucosae in 57% (95%CI 50 to 64) and 61% (95%CI 54 to 68) of biopsies, respectively (OR 1.14 [95%CI 0.77 to 1.69]; *P* = 0.50).

Crush artifacts were rarely observed, regardless of the biopsy method or technique used. Overall, 97% (95%CI 95 to 98) of biopsies were free of crush artifacts. No significant differences were found between the biopsy methods or techniques ( Table 2 ).

However, the percentage of well-oriented biopsies was significantly higher with the single-biopsy method compared with the double-biopsy method (81% [95%CI 75 to 86] vs. 71% [95%CI 64 to 77], OR 1.74 [95%CI 1.08 to 2.78]; *P* = 0.02). The orientation of biopsies was comparable when the advance-and-close and turn-and-suction techniques were used (78% [95%CI 72 to 84] vs. 77% [95%CI 67 to 80], OR 0.77 [95%CI 0.48 to 1.23]; *P* = 0.28) ( Table 2 ).

None of the interaction terms between the co-primary comparisons were significant for any of the histopathological outcome parameters (i.e. presence of muscularis mucosae *P* = 0.24; absence of crush artifacts *P* = 0.35; well-orientated biopsies *P* = 0.96).

Histopathological outcomes per randomization arm are shown in Table 3 .

**Table:** **Table 3** Primary and secondary outcomes for each of the randomization arms (study Part I; prospective factorial design trial). All values represent summary estimates derived from linear or logistic (mixed-effects) regression models corrected for stratification factors and if applicable, repeated measurements.

	Double-biopsy		Single-biopsy	
	Advance-and-close n = 240	Turn-and-suction n = 263	Advance-and-close n = 280	Turn-and-suction n = 241
Primary end point				
Biopsy size, mm ^2^	2.77 (2.44 to 3.09)	2.61 (2.29 to 2.93)	3.14 (2.81 to 3.46)	3.54 (3.22 to 3.86)
Secondary end points				
Histopathological parameters, %				
Presence muscularis mucosae	51 (42 to 61)	60 (51 to 69)	63 (53 to 71)	60 (51 to 69)
Absence of crush artifacts	97 (93 to 99)	95 (90 to 98)	97 (94 to 99)	98 (95 to 99)
Well-orientated biopsies	73 (63 to 81)	68 (58 to 77)	83 (75 to 89)	79 (69 to 86)
Endoscopic parameters				
Biopsy time, seconds	23 (20 to 25)	30 (27 to 32)	24 (22 to 27)	31 (29 to 34)
Retained and lost biopsies, %				
Retained	95 (91 to 98)	94 (89 to 97)	99.7 (98 to 100)	99.6 (98 to 100)
Lost	5 (2 to 9)	6 (3 to 11)	0.3 (0.04 to 2)	0.3 (0.04 to 2)
Data represent mean values (95%CI).				

#### Endoscopic parameters

Overall, the mean biopsy time was 27 seconds per specimen (95%CI 25 to 29). The single-biopsy method did not result in a longer biopsy time than the double-biopsy method. A mean of 28 seconds (95%CI 26 to 30) was needed per biopsy with the single-biopsy method, while 26 seconds per biopsy (95%CI 24 to 28) were required with the double-biopsy method (mean difference 2 seconds per biopsy [95%CI –1 to 4]; *P* = 0.26) ( Table 2 ). In contrast, biopsy time was significantly longer with the turn-and suction technique (31 seconds per biopsy [95%CI 29 to 32]) compared with the advance-and-close technique (24 seconds per biopsy [95%CI 22 to 25]), with a mean difference of 7 seconds per biopsy (95%CI 4 to 10; *P* <0.001).

When the single-biopsy method was used, 0.3% (95%CI 0.07 to 1) of biopsies were lost compared with 6% (95%CI 3 to 11) of biopsies using the double-biopsy method. The OR for the absence of missing biopsies with the single-biopsy vs. double-biopsy methods was 18.1 (95%CI 4.18 to 78.4; *P* <0.001). No significant difference in the number of lost biopsies was observed between biopsy techniques ( Table 2 ).

The interaction terms between the co-primary comparisons were nonsignificant for both endoscopic outcome parameters (i.e. biopsy time *P* = 0.93; lost biopsies *P* = 0.95). Table 3 shows the outcomes of endoscopic parameters per randomization arm.

### Part II – uncontrolled before–after study

A total of 46 patients (288 biopsies) were included before implementation of the single-biopsy method with turn-and-suction technique and 44 patients (256 biopsies) were included after implementation. No significant differences in baseline characteristics were observed ( **Table 2s** ).

Ten endoscopists performed the BE surveillance endoscopies in the pre-implementation period, six of whom (60%) used the advance-and-close technique while four (40%) used the turn-and-suction technique. As for biopsy method, six endoscopists (60%) used the double-biopsy method and four (40%) used the single-biopsy method. Three endoscopists (30%) used the combination of the single-biopsy method with turn-and-suction technique. Hence, 125/288 biopsies (43%) were taken using the single-biopsy method during the pre-implementation phase, while 195/288 biopsies (68%) were taken using the turn-and-suction technique. The combination of both approaches was used in 117/288 biopsies (41%).

Ten endoscopists performed procedures in the post-implementation period, all of whom used the single-biopsy method with turn-and-suction technique (100%). Nine of the 10 endoscopists performed procedures both prior and after the implementation.

Mean biopsy size increased by 18%, from 3.31 mm ^2^ (95%CI 2.95 to 3.68) before implementation to 3.90 mm ^2^ (95%CI 3.50 to 4.29) after implementation (mean difference 0.58 mm ^2^ [95%CI 0.06 to 1.11]; *P* = 0.03) ( Table 4 ). There was no significant increase in mean biopsy size when comparing biopsies taken before and after implementation (n = 117 vs. n = 97) among endoscopists who had already been using the single-biopsy method with turn-and-suction technique prior to implementation (3.72 mm ^2^ [95%CI 2.95 to 4.48] vs. 3.79 mm ^2^ [95%CI 2.97 to 4.62], mean difference 0.08 mm ^2^ [95%CI –1.01 to 1.16]; *P* = 0.88).

**Table:** **Table 4** Mean biopsy size of biopsies taken by nondedicated Barrett esophagus (BE) endoscopists before (n = 288) and after (n = 256) implementation of the single-biopsy method with turn-and-suction technique (study Part II; uncontrolled before–after study). Mean biopsy size was derived from a linear mixed-effects regression model corrected for BE length and repeated measurements.

	Total	Before implementation	After implementation	Mean difference	P
Biopsies, n	544	288	256		
Biopsy size, mean (95%CI), mm ^2^	3.69 (3.42 to 3.97)	3.31 (2.95 to 3.68)	3.90 (3.50 to 4.29)	0.58 (0.06 to 1.11)	0.03

## Discussion

In this multidesign study comprising three separate substudies, the effect of different BE biopsy methods and techniques on both histopathological and endoscopic parameters was evaluated. Our exploratory prestudy revealed that mean biopsy size was 22% larger when the turn-and-suction technique was used instead of the advance-and-close technique, irrespective of the endoscopist’s experience in BE surveillance care. As none of the performing endoscopists reported use of the double-biopsy method, we were unable to evaluate the effect of the single- vs. double-biopsy method on biopsy size. Although the use of retrospective data carries a potential risk of misclassification bias, this analysis supported our hypothesis that differences in biopsy methods or techniques may influence histopathological parameters of biopsy specimens.

Therefore, we conducted a prospective factorial trial, in which we found a mean biopsy size of 2.68 mm ^2^ (95%CI 2.45 to 2.92) in double-method biopsies and 3.34 mm ^2^ (95%CI 3.10 to 3.57) in single-method biopsies ( *P* < 0.001); a difference of 25% in biopsy size. Given the mean of 10 biopsies per procedure, this translates into two additional biopsies in terms of surface area. The turn-and-suction technique did not independently appear superior to the advance-and-close technique in terms of biopsy size, although in the multiarm analysis, biopsies were largest when obtained with the single-biopsy method combined with the turn-and-suction technique (13% larger than biopsies obtained with the single-biopsy method combined with the advance-and-close technique; *P* = 0.08). The interaction term between the co-primary comparisons was nonsignificant ( *P* = 0.08), although this test has low power for detecting true interactions 7 . Therefore, the turn-and-suction technique might be the preferred technique when combined with the single-biopsy method.

With respect to the secondary end points in Part I, biopsies taken with the single-biopsy method were significantly better oriented compared with the double-biopsy method (OR 1.74 [95%CI 1.08 to 2.78]; *P* = 0.02). In addition, we were able to refute the overall assumption that the double-biopsy method decreases total procedure time, as time required to obtain biopsies was comparable for both methods (mean difference of 2 seconds per biopsy [95%CI –1 to 4]; *P* = 0.26). More biopsies were retained when endoscopists used the single-biopsy method instead of the double-biopsy method (OR 18.1 [95%CI 4.18 to 78.4]; *P* <0.001). This could have contributed to the increased biopsy times of the double-biopsy method, though the overall percentage of lost biopsies was relatively small (6%). It will therefore have had only a minor effect on biopsy times. Mean biopsy time was, however, significantly longer when the turn-and-suction technique was used compared with the advance-and-close technique, with a mean difference of 7 seconds per biopsy (95%CI 4 to 10; *P* <0.001). However, when translating this to clinical practice, this would only add approximately 1 minute of procedure time to an average surveillance endoscopy with 10 random biopsies.

In Part II of our study, we implemented the single-biopsy method with the turn-and-suction technique in routine clinical practice at one of the participating hospitals in which participating endoscopists mainly used the double-biopsy method and advance-and-close technique. Mean biopsy size increased by 18% after implementation ( *P* = 0.03), which endorses our findings in our exploratory prestudy and factorial design trial.

Few prospective studies have been performed to compare the double- vs. single-biopsy methods and advance-and-close vs. turn-and-suction techniques, mostly with a different origin of biopsies and with alternative definitions of biopsy size, thereby hampering a direct comparison with our study results 5 8 9 10 11 . However, in concordance with our results, Hookey et al. 10 (colonic biopsies) and Padda et al. 8 (gastric and normal-lining esophageal biopsies) demonstrated higher percentages of lost biopsies when using the double-biopsy method. In addition, Latorre et al. 9 (duodenal biopsies) confirmed significantly higher percentages of well-oriented biopsies with the single-biopsy method compared with the double-biopsy method.

Apart from histopathological parameters, the turn-and-suction technique involves directing the endoscope toward the biopsy area of interest with the biopsy forceps cups placed against the tip of the endoscope. Consequently, compared with the advance-and-close technique, the turn-and-suction technique may result in the acquisition of biopsies in a more controlled fashion and could be considered in cases with visible lesions requiring targeted biopsies.

The strengths of the current study are its multidesign approach and the evaluation of different biopsy methods and techniques on both histopathological parameters and endoscopic parameters. Furthermore, the presence of a research fellow or research nurse during the endoscopies in the prospective factorial trial ensured adequate data collection.

This study also has limitations that are noteworthy. Most importantly, as no validated criteria for histopathological quality of BE biopsy specimens exist, the choice for our primary and secondary outcomes was based on expert opinion. Even though it was anticipated that increased biopsy size leads to increased biopsy quality, the extent to which this surrogate end point affects clinically relevant outcomes such as dysplasia detection or interobserver variability among pathologists remains unclear. However, a trial using these outcome parameters would require an immense number of biopsies. In addition, biopsy size was defined as surface area of the entire biopsy specimen. We made no distinction in epithelial and subepithelial surface area, despite the epithelium being of particular interest in BE biopsies. Next, various types of biopsy forceps are available but only two were used in our study. Although not part of the study, we acknowledge the choice of biopsy forceps may influence biopsy size as well as other histopathological parameters 12 13 . Finally, our factorial trial was not powered for multiplicity adjustments in co-primary comparisons. Such adjustments in factorial trials are debated 14 15 16 17 , meaning results with *P* values between 0.025 and 0.05 should be interpreted with caution. However, none of the *P* values in this part of our multidesign study fell within this range.

In conclusion, our study provides evidence for the use of the single-biopsy method combined with the turn-and-suction technique as the preferred method and technique for obtaining biopsy samples in patients with BE.

## Supplementary Material

Supplementary Material Zusatzmaterial
