# Supplementary material for: Endoscopic biopsy techniques in Barrett esophagus patients: a multidesign study
Source: Endoscopy. 2025 Jun 24;57(9):962–71. doi: 10.1055/a-2606-7682 (PMC12417858; doi:10.1055/a-2606-7682)

Supplementary material. Beaufort IN, Elias SG, Akkerman EMP, et al.

Endoscopic biopsy techniques in Barrett's esophagus patients: a multidesign study

Supplementary Methods and Results

Methods and results of the exploratory pre-study

Methods

A retrospective cohort analysis was performed to compare BE biopsies obtained by endoscopists with different levels of expertise in BE surveillance care (i.e. dedicated BE endoscopists and non-dedicated BE endoscopists) using different biopsy methods and techniques. This study had an exploratory nature and was performed in one teaching hospital (St. Antonius Hospital) between October 2019 and March 2021.

Adult patients with a diagnosis of BE <10cm in maximum length, scheduled for a surveillance endoscopy with random biopsies in the aforementioned time frame were eligible. A random sample of 65 eligible patients was selected for further analysis; 25 and 40 patients in whom endoscopies were performed by dedicated BE endoscopists and non-dedicated BE endoscopists, respectively.

All random biopsies of the included patients were independently analyzed on biopsy size by two members of the research team (I.B. and E.A.), with biopsy size defined as surface area in mm^2. If a difference >10% was found between the two surface area measurements of a single biopsy specimen, the measurement was repeated in the presence of both researchers. All biopsies were obtained using the same biopsy forceps (Endo Jaw FB220U; Olympus, Tokyo, Japan).

In addition, the performing endoscopists were asked to fill out a short questionnaire regarding their preferred biopsy method and technique. Both assessors of biopsy size were blinded to the questionnaire results.

Primary endpoint was the size of biopsy specimens in mm^2. Mixed effects linear regression analyses (identity link function; restricted maximum likelihood estimation) with a random intercept per patient were used to assess differences in biopsy size. Biopsy method, biopsy technique, BE length, and type of endoscopist (i.e. dedicated BE endoscopist vs. non-dedicated BE endoscopist) were included as fixed effects. The intraclass correlation coefficient was (ICC) was used to assess the interobserver agreement. ICC values less than 0.5 were interpreted as poor agreement, 0.5–0.75 as moderate agreement, 0.75–0.9 as good agreement, and >0.90 as excellent agreement [1]. Data were analyzed in R version 3.5.1 for Windows. The significance level was set at $P < 0.05$.

Supplementary material. Beaufort IN, Elias SG, Akkerman EMP, et al.
Endoscopic biopsy techniques in Barrett's esophagus patients: a multidesign study

Results

In total, 478 random biopsies of 65 BE patients were obtained by 17 different endoscopists; 2 dedicated BE endoscopists and 15 non-dedicated BE endoscopists. Baseline characteristics of the included patients and their endoscopies are presented in **Table 1s**.

All 17 endoscopists (17/17; 100%) reported to use the single-biopsy method; 11 endoscopists (11/17; 65%) used the turn-and-suction technique. Consequently, 72/478 biopsies (15%) of 14 patients were taken with the advance-and-close technique compared with 406/478 biopsies (85%) of 51 patients taken with the turn-and-suction technique.

Biopsies were significantly larger when obtained with the turn-and-suction technique (3.83 mm^2 [95%CI 3.57 to 4.09]) instead of the advance-and-close technique (3.13 mm^2 [95%CI 2.54 to 3.72], mean difference 0.70 mm^2 [95%CI 0.05 to 1.35]; $P = 0.036$), translating into an increase of 22%. In contrast, mean biopsy size did not significantly differ between biopsies obtained by non-dedicated BE endoscopists vs. dedicated BE endoscopists (3.87 mm^2 [95%CI 3.54 to 4.20] vs. 3.58 mm^2 [95%CI 3.22 to 3.95], mean difference 0.28 mm^2 [95%CI –0.23 to 0.80]; $P = 0.27$). Among biopsies taken by non-dedicated BE endoscopists only (n = 236), biopsies were 23% larger if obtained with the turn-and-suction technique compared with the advance-and-close technique (3.99 mm^2 [95%CI 3.56 to 4.43] vs. 3.25 mm^2 [95%CI 2.65 to 3.86], mean difference 0.74 mm^2 [95%CI –0.003 to 1.49]; $P = 0.051$).

The interobserver agreement in measuring biopsy size was excellent (ICC 0.998 [95%CI 0.997 to 0.998]).

<u>Reference</u>

1 Koo TK, Li MY. A Guideline of Selecting and Reporting Intraclass Correlation Coefficients for Reliability Research. J Chiropr Med 2016; 15: 155–163

Thieme

Supplementary material. Beaufort IN, Elias SG, Akkerman EMP, et al.

Endoscopic biopsy techniques in Barrett's esophagus patients: a multidesign study

Table 1s Baseline characteristics of the included BE patients in the exploratory pre-study (i.e. retrospective analysis). All patients underwent surveillance endoscopies in one teaching hospital between October 2019 and March 2021.

	**Total**
**Number of patients**	65
**Age**, mean (SD)	65 (11)
**Male**, n (%)	48 (74)
**ASA classification**, n (%)	
I	16 (25)
II	40 (62)
III	7 (11)
Unknown	2 (3)
**Hiatal hernia**, cm, median (IQI)	2 (1-4)
**BE length**, cm, median (IQI)	
Circumferential	0 (0-2)
Maximum	3 (2-4)
**Maximum BE length**, n (%)	
≤ 3 centimeter	37 (57)
> 3 centimeter	28 (43)
**Esophagitis**, n (%)	6 (9)
**Sedation**, n (%)	
No sedation	16 (25)
Midazolam	43 (66)
Propofol	6 (9)
**Endoscopist**, n (%)	
Dedicated BE endoscopist	25 (38)
Non-dedicated BE endoscopist	40 (62)

Abbreviations: BE, Barrett esophagus; IQI, interquartile interval; SD, standard deviation.

Supplementary material. Beaufort IN, Elias SG, Akkerman EMP, et al.

Endoscopic biopsy techniques in Barrett's esophagus patients: a multidesign study

Table 2s Baseline characteristics of the included BE patients undergoing surveillance endoscopies at Diakonessenhuis before and after the implementation of the single-biopsy turn-and-suction technique in study Part II (i.e. uncontrolled before–after study). All endoscopies were performed by non-dedicated BE endoscopists.

	Total	Before implementation	After implementation	p-value
**Number of patients**	90	46	44	
**Age**, mean (SD)	65 (10)	65 (11)	65 (8)	0.67
**Male**, n (%)	64 (71)	31 (67)	33 (75)	0.57
**ASA classification**, n (%)				
I	16 (18)	6 (13)	10 (23)	
II	62 (69)	33 (72)	29 (66)	
III	11 (12)	6 (13)	5 (11)	
Unknown	1 (1)	1 (2)	0 (0)	0.51
**Hiatal hernia**, cm, median (IQI)	3 (2-4)	3 (2-5)	2 (2-4)	0.43
**BE length**, cm, median (IQI)				
Circumferential	1 (0-3)	2 (1-3)	1 (0-2)	0.05
Maximum	3 (2-5)	3 (2-5)	2 (2-4)	0.08
**Maximum BE length**, n (%)				
≤ 3 centimeter	54 (60)	24 (52)	30 (68)	
> 3 centimeter	36 (40)	22 (48)	14 (32)	0.18
**Esophagitis**, n (%)	10 (11)	8 (9)	2 (2)	0.11
**Sedation**, n (%)				
No sedation	19 (21)	9 (20)	10 (23)	
Midazolam	67 (74)	37 (80)	30 (68)	
Propofol	4 (4)	0 (0)	4 (9)	0.09
**Biopsy method**, n (%)				
Double-biopsy	29 (32)	29 (63)	0 (0)	
Single-biopsy	61 (68)	17 (37)	44 (100)	NA
**Biopsy technique**, n (%)				
Advance-and-close	17 (19)	17 (37)	0 (0)	
Turn-and-suction	73 (81)	29 (63)	44 (100)	NA

Abbreviations: BE, Barrett esophagus; IQI, interquartile interval; SD, standard deviation

Supplementary material. Beaufort IN, Elias SG, Akkerman EMP, et al.

Endoscopic biopsy techniques in Barrett's esophagus patients: a multidesign study

Fig. 1s A schematic illustration of the advance-and-close technique and the turn-and-suction technique. **A.** The biopsy forceps is placed in front of the Barrett's esophagus target area. **B.** The advance-and-close technique: the forceps cups are pressed into the mucosa. **C**. The turn-and-suction technique: suction is applied, causing the mucosa to collapse into the cups

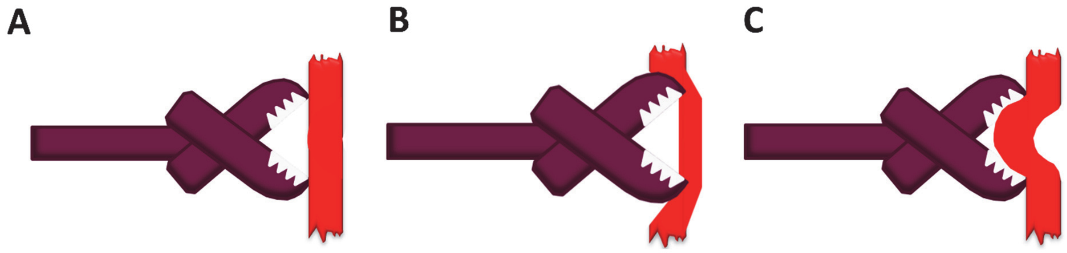

Fig. 2s Examples of biopsies taken from Barrett's esophagus mucosa. **A.** A well-oriented biopsy containing muscularis mucosae (black dotted lines). **B.** An insufficiently orientated biopsy without evident muscularis mucosae. Instead, only small strands of muscularis mucosae are present (black arrow).

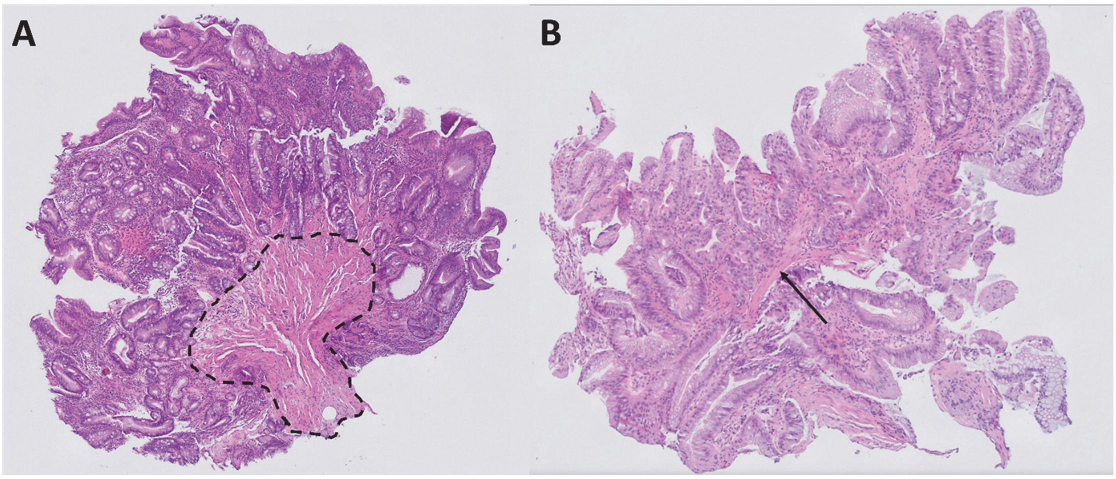

Supplementary material. Beaufort IN, Elias SG, Akkerman EMP, et al.

Endoscopic biopsy techniques in Barrett's esophagus patients: a multidesign study

Fig. 3s A violin plot with a boxplot overlay depicting the distribution of biopsy size for each biopsy method in study Part I (i.e. prospective factorial design trial). The overall shape represents the density of the data across the range of values. The horizontal line within the boxplot represents the median biopsy size, whereas the box represents the 25th and 75th percentiles. The whiskers indicate values within 1.5 times the interquartile range and the dots note outliers.

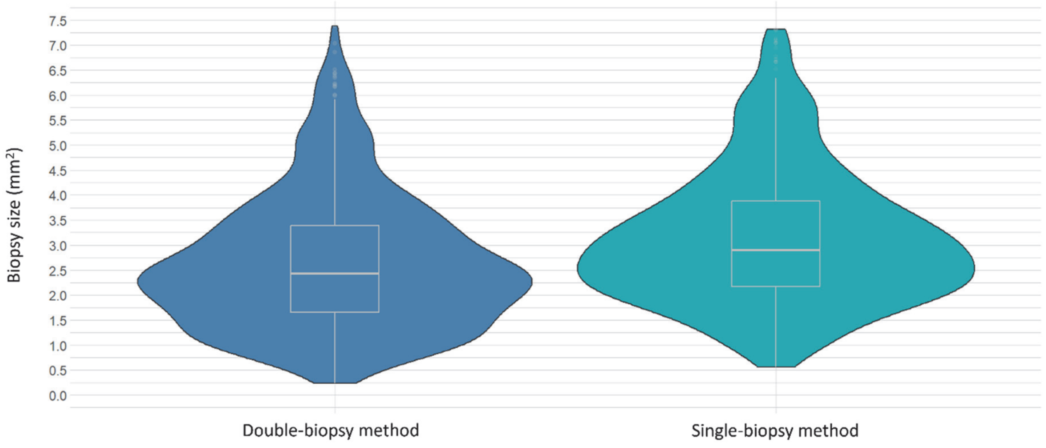

Fig. 4s A violin plot with a boxplot overlay depicting the distribution of biopsy size for each biopsy technique in study Part I (i.e. prospective factorial design trial). The overall shape represents the density of the data across the range of values. The horizontal line within the boxplot represents the median biopsy size, whereas the box represents the 25th and 75th percentiles. The whiskers indicate values within 1.5 times the interquartile range and the dots note outliers.

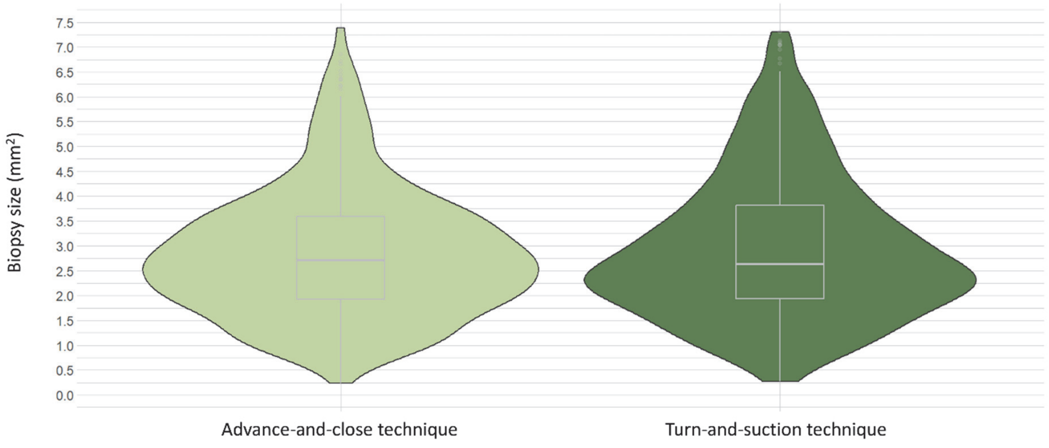

Supplement: Supplementary file 2 — Supplementary Material [file 10-1055-a-2606-7682_26082637.pdf]
